# Co-occurrence of malaria and Chagas disease in the Brazilian Amazon: the need for integrated health surveillance

**DOI:** 10.1590/0102-311XEN042124

**Published:** 2025-06-09

**Authors:** Izabel Cristina dos Reis, Raquel Martins Lana, Cláudia Torres Codeço, Ana Paula Dal’Asta, Milton Barbosa, Diego Ricardo Xavier

**Affiliations:** 1 Instituto de Comunicação e Informação Científica e Tecnológica em Saúde, Fundação Oswaldo Cruz, Rio de Janeiro, Brasil.; 2 Centro Nacional de Supercomputación, Barcelona, España.; 3 Programa de Computação Científica, Fundação Oswaldo Cruz, Rio de Janeiro, Brasil.; 4 Instituto Nacional de Pesquisas Espaciais, São José dos Campos, Brasil.; 5 Laboratório de Ecologia Evolutiva e Biodiversidade, Universidade Federal de Minas Gerais, Belo Horizonte, Brasil.

**Keywords:** Malaria, Chagas Disease, Integrality in Health, Decision Trees, Malaria, Enfermedad de Chagas, Integralidad en Salud, Árboles de Decisión

## Abstract

This study addresses the co-occurrence of malaria and Chagas disease in municipalities in the Amazon, a region characterized by geographic and climatic diversity and by socioeconomic and environmental transformations. This study aimed to identify the factors related to the co-occurrence of malaria and Chagas disease in the Brazilian Amazon from 2015 to 2019. The analysis explored 19 environmental indicators and two socioeconomic indicators related to habitat loss, land use and cover, climate anomalies, and the multidimensional poverty index. Modeling was performed by Conditional Inference Trees, adjusting models with and without contextual variables, to map areas of probable co-occurrence of the diseases. The incidence of malaria is predominant in the western Amazon, while Chagas disease is more frequent in areas of Pará and parts of Amazonas and Acre. Municipalities with high coverage of native vegetation showed higher incidences of malaria, but not necessarily of Chagas disease. Municipalities with native vegetation cover and pasture areas showed heterogeneous incidence of diseases, with some presenting a high incidence of both diseases. The predictive analysis showed an increase in the number of municipalities with a high expected incidence of malaria (moderate) and disease Chagas (high) from 1 to 7, when compared to observed data. The study showed areas with a risk of moderate and high incidence of both diseases, covering a larger region than that observed in the period. Alternatives of shared surveillance and the integration of programs for the identification of cases and treatment can be a measure to optimize resources and help eradicate these diseases in the region.

## Resumen

Este estudio aborda la coocurrencia de la malaria y la enfermedad de Chagas en los municipios amazónicos, una región caracterizada por la diversidad geográfica y climática, y por las transformaciones socioeconómicas y ambientales. El objetivo de este estudio fue identificar los factores relacionados con la coocurrencia de la malaria y de enfermedad de Chagas en la Amazonía brasileña en el período comprendido entre 2015 y 2019. El análisis exploró 19 indicadores ambientales y dos socioeconómicos relacionados con la pérdida de hábitat, con el uso del suelo y su cobertura, con las anomalías climáticas y con el índice de pobreza multidimensional. El modelado fue realizado por Árboles de Regresión con Inferencia Condicional, ajustando modelos con variables contextuales y sin ellas para mapear probables zonas de coocurrencia de enfermedades. La incidencia de malaria predomina en la Amazonía occidental, mientras que la enfermedad de Chagas tiene una mayor concentración en las zonas de Pará y partes de Amazonas y Acre. Los municipios con alta cobertura de vegetación nativa mostraron una mayor incidencia de malaria, pero no necesariamente de Chagas. Los municipios con cobertura vegetal nativa y áreas de pastoreo presentaron una heterogeneidad en la incidencia de enfermedades, y algunos tuvieron una alta incidencia de ambas enfermedades. El análisis predictivo pasó de 1 a 7 el número de municipios con alta incidencia estimada de malaria (promedio) y de enfermedad de Chagas (alto) en comparación con los datos observados. Los análisis indican zonas de riesgo de incidencia mediana y alta de ambas enfermedades al cubrir una región más grande que la observada en el período. Las alternativas de vigilancia compartida y la integración de programas de identificación y tratamiento de casos pueden ser una forma de optimizar recursos y buscar erradicar estas enfermedades en la región.

## Introduction

The Brazilian Amazon is a complex and dynamic territory with a variety of ethnic groups, landscapes, and ecosystems. It includes a large part of the Amazon rainforest, the largest reserve of biological species diversity in the world, with a vast amount of natural resources and a crucial role in regulating local and regional climate patterns ^1^ ^,^ ^2^. Since 2012, there has been a progressive increase in deforestation rates, with a peak in 2021, when it reached more than 13,000km^2^ of Amazon rainforest were destroyed ^3^. It is estimated that around half (2.5 million km^2^) of this forest is currently degraded ^4^.

This situation has directly and indirectly influenced the dynamics of diseases transmitted by insect vectors, which have a significant socioeconomic and health impact on local population, especially those people exposed to precarious housing and working conditions ^5^. Environmental changes tend to change the ecological balance and the context in which pathogens, parasites and their vectors and hosts develop and transmit diseases ^6^. 

Several vector-borne diseases affect the population in the Amazon, including malaria and Chagas disease. Malaria, one of the most prevalent infectious diseases in the Amazon, is caused by parasites of the genus *Plasmodium* and transmitted to humans through the bite of infected female mosquitoes of the genus *Anopheles*. From 2013 to 2023, 1,642,714 cases were reported in the Amazon Region, most of them caused by *P. vivax* (86%) ^7^. *A. darlingi* is the main vector, but other species are also considered as malaria vectors in the region ^8^. The dynamics of malaria transmission in areas of extractive activities, agricultural zones, urban and suburban areas, and indigenous reserves are distinct, since they involve human populations with specific demographic, social, and economic characteristics ^9^ and differentiated landscapes that determine distinct ecological factors and respective communities of *Anopheles* ^10^. Deforestation and mining are some of the main challenges of the malaria eradication program in the Amazon Region ^11^.

Chagas disease is caused by *Trypanosoma cruzi* and transmitted to humans by triatomines of the genera *Triatoma*, *Rhodnius*, and *Panstrongylus*. In Brazil, the transmission of Chagas disease through the consumption of contaminated açaí juice, which is processed in an artisanal manner without heat treatment, has been increasingly reported ^12^. From 2010 to 2020, Brazil reported 2,777 acute cases of Chagas disease; 84% of these cases were reported in the North, with 1,996 acute cases in the State of Pará ^13^. The progressive migration of people and domestic animals from rural to urban areas and the adaptation of vectors to the outskirts of cities due to displacement resulting from deforestation or urbanization have favored the maintenance of the Chagas disease transmission cycle, mainly through the consumption of contaminated food ^14^ ^,^ ^15^. 

The detection of *T. cruzi* in thick smears of asymptomatic and symptomatic malaria patients, a strategy implemented in 2008, has increased the sensitivity of the detection of the occurrence or co-occurrence and coinfections of these two diseases ^16^ ^,^ ^17^. This overlap offers potential synergies and opportunities for the use of common resources in an integrated public health approach to both diseases. In this context, this study addresses the co-occurrence of malaria and Chagas disease in the same population, without investigating the coinfection between their etiological agents, which, despite representing a risk in this scenario, occurs in only 0.2% of confirmed malaria cases ^7^. Co-occurrence represents a substantial challenge for Public Health in the Amazon, demanding surveillance, diagnosis, and control strategies that consider the complex ecological and social interactions of the region. Integrated surveillance is essential in this context, as it promotes the collaboration of different control programs, sectors, and communities, using data to monitor and respond efficiently to outbreaks and disease spread ^18^. Therefore, the objective of this study was to identify the environmental and social factors related to the co-occurrence of malaria and Chagas disease in the Brazilian Amazon with a view to integrated public health surveillance.

## Method

### Study site

The Legal Amazon, a political-administrative region, has around 5 million km^2^, representing about 59% of the Brazilian territory. This region covers nine states distributed in different geographic regions of the country: Acre, Amapá, Amazonas, Pará, Rondônia, Roraima, and Tocantins in the North Region; part of Maranhão in the Northeast Region; and Mato Grosso in the Central West Region, totaling 772 municipalities and about 18 million inhabitants ^19^, and presenting significant geographic and administrative diversity.

Geographically located in the equatorial zone, the study area is characterized by a predominantly hot and humid climate ^20^, marked by a rainy season and a dry season. The intensity and duration of these periods may vary in each municipality/location. 

### Data and variables

The harmonized database of the Brazilian Legal Amazon named Trajetórias dataset ^21^ (https://zenodo.org/records/7098053) was used. This database contains a series of environmental, epidemiological, and socioeconomic indicators at the municipal level for the first two decades of the 21st century. This study used data related to the total number of reported cases of malaria (*vivax*, *falciparum*, and mixed) and acute Chagas disease from 2015 to 2019, by rural and urban area of each municipality. The cases of malaria caused by all species and from both rural and urban areas were added to obtain the total number of cases per municipality in the 5-year period. The population size used to calculate the epidemiological indicators was that for 2017, available in the Trajetórias dataset. Based on the total number of cases, the annual parasite index (API) was calculated for the period analyzed for each municipality, as described in Rorato et al. ^21^. The API is the most widely used indicator to measure the number of confirmed malaria cases in each municipality divided by the population of the municipality during the study period, multiplied by 1,000. It does not measure the incidence because of the uncertainty in the attribution of a new case or relapse due to *vivax* malaria ^22^; however, for comparison purposes with Chagas disease, we will call it incidence. The incidence of disease Chagas was calculated using the same mathematical expression, with the total number of new acute cases in the numerator and the same denominator used for malaria.

In order to identify environmental factors associated with the co-occurrence of the two diseases, 19 environmental indicators from the Trajetórias dataset were used. Of these, seven describe the habitat loss observed between 2010 and 2017 due to the loss of forest cover, that is, deforestation in the period in relation to the original forest (*deorg*) and in relation to the remaining forest at the beginning of the period (*defor*), forest fire (*fire*), and the gradual loss of forest by selective logging and/or fire (degradation of the original forest - *dgorg* - and recent degradation - *dgfor*), in addition to two measures of spatial fragmentation of areas with natural vegetation cover regarding the core area (*core*) and edge area (*edge*). Six other indicators describe the footprints left on the landscape by production systems, based on land use and land cover classes mapped in 2017 (proportion of pasture - *pasture,* remaining forest - *refor*, secondary vegetation - *secveg*, crop - *crop*, mining - *mining*, and urbanized areas - *urban*). Three indicators describe mobility and connectivity by measuring the density of transportation networks in 2017 (river, ports, and roads). Finally, three indicators measure the spatial magnitude of climate anomalies from 2007 to 2017: precipitation (*precp* - positive anomaly; *precn* - negative anomaly) and temperature (tempp - positive temperature anomaly). Of all socioeconomic indicators available in the database, only the multidimensional poverty index (MPI) of 2010 was used for the rural (*ipm_rural*) and urban (*ipm_urb*) strata of each municipality. The MPI is calculated by multiplying the incidence of poverty by the average intensity of poverty in the dimensions of health, education, and living conditions. All these 21 indicators were used without additional processing. Some of the environmental indicators from the Trajetórias dataset were calculated using data from the PRODES project (Satellite Monitoring of Deforestation in the Brazilian Amazon Forest), which monitors clear cutting in areas of primary forest vegetation type in the Legal Amazon. Then, for the municipalities that are included in the Legal Amazon, but with a dominant vegetation type that differs from the forest vegetation type, the indicators associated with this coverage (*deorg*, *defor*, *dgorg*, and *dgfor*) were not calculated. Details on the construction and calculation of each indicator are found in Rorato et al. ^21^.

### Data analysis

#### Identification of co-occurrence areas

**Figure 1:**
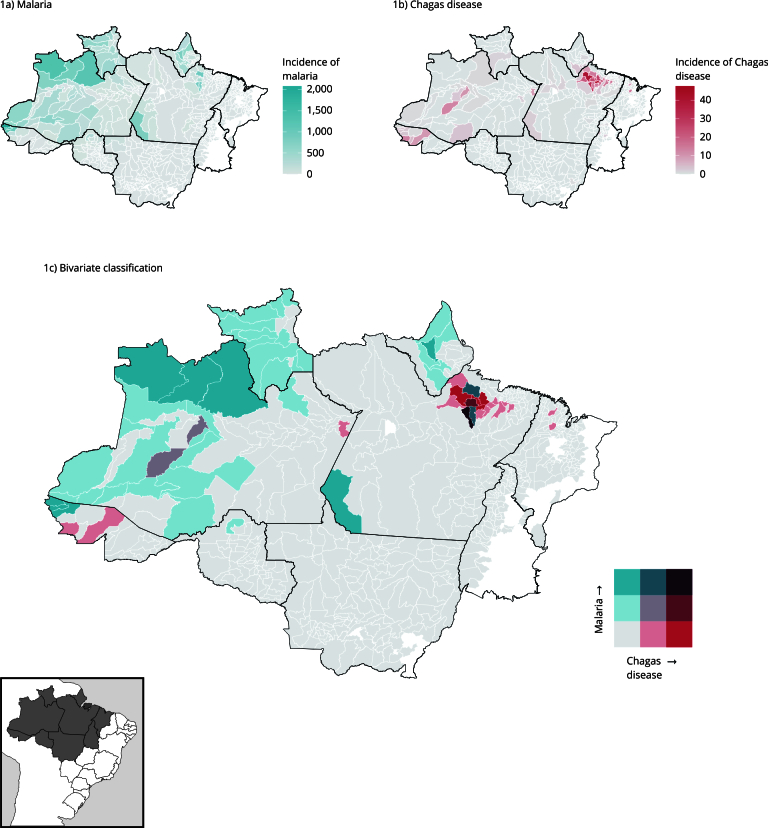
Incidence of malaria and Chagas disease from 2015 to 2019, in municipalities in the Brazilian Amazon, bivariate classification of the two diseases by the Jenks method.

Note: the legend in Figure 1c indicates the incidence of disease Chagas in shades of red and the incidence of malaria in shades of blue. The diagonal colors indicate a gradient of co-occurrence.

To identify the co-occurrence areas of malaria and Chagas disease in the Legal Amazon, bivariate mapping was performed with the Jenks ^23^ method, using the incidence of both diseases as outcomes. This method is frequently used in the analysis of geographic data to group spatial data in a way that minimizes the variation within each group and maximizes the variation between groups. In this study, the Jenks ^23^ distribution in three dimensions (high, moderate, and low) was considered for the two diseases analyzed. The intersection of these dimensions generated nine classes, represented in a matrix whose diagonal represents low incidence for both diseases in the lower left position and high incidence in the upper right position (see legends in Figure 1). The analysis was performed using the *{biscale}* ^24^ library of the R software tool (http://www.r-project.org).

#### Socio-environmental modeling

In order to map areas of probable co-occurrence of the two diseases (outcome variables), multivariate classification models were adjusted - the first without covariates and the second model with environmental and social contextual variables. The socioenvironmental classification model is a Conditional Inference Tree (CIT) type, with three primary levels of results consisting of root node, internal nodes, and terminal nodes (leaf nodes) ^25^. The model is implemented in the *{partykit::ctree()}* ^26^ library of the R software tool. In epidemiology, CIT is a suitable method to handle complex datasets and identify interactions between multiple factors that affect population health ^27^. CIT uses a non-parametric machine learning technique that considers the partition criterion based on statistical significance and evaluates the conditional relationships between contextual variables in search of homogeneous groups that describe the distribution of the study variable. The algorithm does not present bias in variable selection and is can handle both numerical and categorical data ^28^.

The algorithm starts by testing the global null hypothesis of independence between any of the contextual variables and the outcome variable; if the hypothesis cannot be rejected, the partition is interrupted. If this hypothesis cannot be rejected, the covariate presenting the strongest association with the outcome of interest is selected as a candidate for splitting, through statistical hypothesis testing and its p-value. If the minimum p-value is greater than the significance threshold adjusted for multiple testing, no variable is selected for splitting and the node is considered a terminal node ^26^ ^,^ ^29^.

In our study, two parameters are highlighted for the construction of the socio-environmental model. The *testtype* parameter of Monte-Carlo simulations that calculates the distribution of the statistical test that considers the sum of the squares of the residuals was used to assess whether there is a significant association between the socio-environmental covariates and the incidence of malaria and Chagas disease. The mincriterion parameter determines the significance threshold for a splitting in the CIT, and was set at 0.99 (p-value < 0.001), which resulted in a more conservative CIT with fewer participations. The test statistics generated through the Monte-Carlo simulations is then compared to the *mincriterion* to decide whether to perform a splitting. Based on these results, the partitions were performed and the groups were created with the predicted values of the outcome variables for the terminal nodes. The calculation of means and confidence intervals of the explanatory variables and the mean square errors (MSE) of the predicted values in the groups (terminal nodes) are presented in the Supplementary Material (https://cadernos.ensp.fiocruz.br/static//arquivo/suppl-e00042124-ing_6198.pdf), and were performed using the non-parametric method of *bootstrap* with a resampling parameter equal to 1,000 using the *{boot}* library ^30^. Finally, the values estimated by the socio-environmental model were again plotted using the bivariate function with the Jenks distribution to map the predicted risk of co-occurrence of malaria and Chagas disease.

In all analyses, only municipalities with complete data for all indicators were considered, resulting in 652 municipalities (84.5%) out of a total of 772. The excluded municipalities are those recently created or that did not present data for the calculation of some environmental indicators ^21^.

## Results

Figure 1 shows the distribution of malaria and Chagas disease incidences from 2015 to 2019 in the 652 municipalities studied in the Brazilian Amazon and the joint spatial distribution of the two variables according to the Jenks distribution. Malaria occurred with higher incidence mainly in the Western Amazon, in the municipalities of Mâncio Lima, Rodrigues Alves, and Cruzeiro do Sul in Acre; São Gabriel da Cachoeira, Barcelos, and Santa Isabel do Rio Negro in Amazonas. In Eastern Amazon, malaria predominated in Anajás, Bagre, and Jacareacanga in Pará; and in Serra do Navio and Calçoene in Amapá (Figure 1a). Chagas disease had a more concentrated distribution, with the highest incidences in the northeast region of Pará, especially in the municipalities of Limoeiro do Ajuru, Breves, Bagre, São Sebastião da Boa Vista, and Muaná; and some municipalities in Amazonas and Acre (Figure 1b).

When the co-occurrence profile was analyzed using the Jenks method (Figure 1c), only one municipality (Bagre, Pará State) was classified as high incidence of both diseases. Among the municipalities with a moderate to high co-occurrence, one municipality (Curralinho, Pará State) showed a high incidence of Chagas disease and a moderate incidence of malaria, and two municipalities (Oeiras and Anajás, Pará State) had a high incidence of malaria and a moderate incidence of Chagas disease. These four municipalities are located on the Marajó Island (Curralinho and Anajás) and in the Lower Tocantins River region (Oeiras and Bagre, Pará State). The municipalities with a moderate incidence for both diseases include Carauari and Uarini, both in the geographical region of Tefé in Amazonas. Finally, 18 municipalities (13 in Pará, 2 in Acre and Maranhão, and 1 in Amazonas) showed a moderate (n = 14) to high (n = 4) incidence of Chagas disease, but a low incidence of malaria; and 39 municipalities (17 municipalities in Amazonas, 11 in Roraima, 6 in Amapá, 3 in Acre, 1 in Pará, and 1 in Rondônia) had a moderate (n = 31) to high (n = 8) incidence of malaria, but a low incidence of Chagas disease.

The adjusted CIT model showed seven explanatory variables for the level of co-occurrence of the two diseases. Among the environmental variables, the following are important predictors: *core*, *pasture*, *edge*, *urban*, and *secveg*. Among the forest transformation variables, deorg was an important variable. And among the socioeconomic indicators, *ipm_urb* was the most important.

### Municipalities with high native vegetation coverage

**Figure 2:**
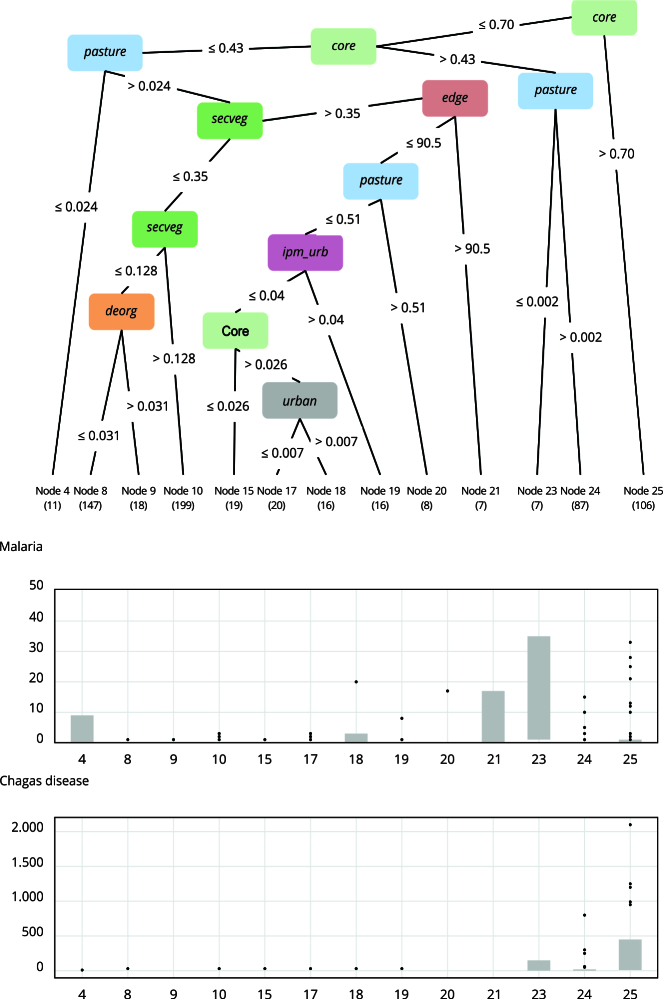
Classification of municipalities according to the Conditional Inference Tree (CIT) model and spatial distribution of the terminal nodes for variables in the Trajetórias dataset.

Figure 2 shows the graphical illustration of the CIT model. It is a tree with 13 terminal nodes, of which three associate municipalities with malaria: 25, 23, and 24. The main predictor variable for malaria was high forest coverage. Municipalities with core area > 70.6% had the highest incidence of malaria. This variable showed a direct relationship with the occurrence of the disease, classifying 16.3% of the municipalities (node 25), including Cruzeiro do Sul, Rodrigues Alves, and Mâncio Lima in Acre, and São Gabriel da Cachoeira and Barcelos in Amazonas, all in the Eastern Amazon. Regarding Chagas disease, the municipalities of Curralinho and Anajás on the Marajó Island, and Bagre in the Lower Tocantins region of Pará, which had been identified by the Jenks method with high rates of both Chagas disease and malaria, are also included in node 25. However, except for a few municipalities, most municipalities in this high vegetation category do not report a high incidence of Chagas disease.

### Municipalities with moderate native vegetation coverage

The second most important predictor variable was the area of the municipality covered by pasture. In 94 municipalities (13.3%) with intermediate to high native vegetation coverage (core area between 43% and 70.6% of the municipal area), the pasture variable was important to identify the co-occurrence of malaria and Chagas disease. Absence of pasture, defined here as pasture < 0.2%, was strongly associated with the presence of Chagas disease (node 23). This condition was found in 7 municipalities (1.7%), including Limoeiro do Ajuru and Breves in the State of Pará, with a moderate incidence of malaria through the Jenks method. In the remaining 87 municipalities, with presence of pasture (pasture > 0.2%), there is greater heterogeneity in the incidence of both diseases (node 24). However, some important municipalities with high incidence of both diseases are identified in this node, such as Oeiras do Pará in Pará, and municipalities with a high incidence of malaria in Cantá in Roraima and Candeias do Jamari in Rondônia, and a high incidence of Chagas disease in Muaná in Pará and Barreirinhas in Amazonas. In addition, some capitals such as Porto Velho (Rondônia) and Manaus (Amazonas) are also classified in node 24.

### Municipalities with low native vegetation coverage

In municipalities with low native vegetation coverage (*core* < 43%), the occurrence of malaria is absent or low. In these conditions, the occurrence of Chagas disease is generally associated with low pasture coverage (< 2.4% of the territory) (node 4). In this group, the highest incidence of malaria is observed in Cametá in Pará, where a moderate incidence of Chagas disease is found. The highest incidence of Chagas disease is observed in Igarapé-Miri in Pará, where the incidence of malaria is low. Both municipalities are neighbors, located in the Lower Tocantins region. 

In the remaining municipalities, characterized by a high presence of pasture (*pasture* > 2.4%), 7 municipalities have a moderate incidence of Chagas disease (node 21). These municipalities have heterogeneous landscapes, where the remaining natural vegetation is very fragmented (*core* area < 46% and *edge* area > 90,514), with significant secondary vegetation coverage (> 35%), and pastures which account for more than 2.4% of the municipal area. The municipalities with the highest incidence in this group are Acará, Bujaru, and São Domingos do Capim, all in Pará.

### Municipalities with heterogeneous landscapes

The inclusion of other variables (*defor*, *deorg*, *urban*, and *ipm-urban*) helped identify more diverse environmental profiles associated with the presence of disease Chagas. Nodes 8 in 147 municipalities (22.5%) and node 9 in 18 municipalities (2.8%) include the variable deorg that measures deforestation in relation to the area originally covered by forest. With a cutoff point of 3.1%, municipalities are classified as presenting a low incidence of malaria and Chagas disease and many municipalities with no cases reported during the period. Most of these municipalities are located in Mato Grosso, Maranhão, Tocantins, and Pará. Nodes 15, 17, 18, and 19 include 62 municipalities (9.5%) and are defined by the variables *imp_urb* and urban - the first referring to the incidence of urban multidimensional poverty and the second to the coverage of urbanized areas. These municipalities presented a low incidence of malaria, however the classification of the municipalities of Abaetetuba in Pará (node 18) and Turilândia in Maranhão (node 19) should be highlighted as they have a high rate of Chagas disease.

Considering the groups in node 25, of the 106 classified municipalities, 98 presented variations when comparing the classification obtained by the Jenks method with that resulting from the application of the CIT model. Such variations indicate a significant influence of the predictor variables. The municipalities that maintained their classifications in both analyses include Rodrigues Alves, Cruzeiro do Sul, and Mâncio Lima in Acre; Barcelos, São Gabriel da Cachoeira, and Santa Isabel do Rio Negro in Amazonas; and Jacareacanga in Pará, which remained with a high incidence of malaria and a low incidence of Chagas disease. On the other hand, in node 24, Candeias do Jamari in Rondônia and Cantá in Roraima remained in the group with a low incidence of Chagas disease and a moderate incidence of malaria. Most of the other municipalities were previously classified as having low incidences for both diseases.

**Figure 3:**
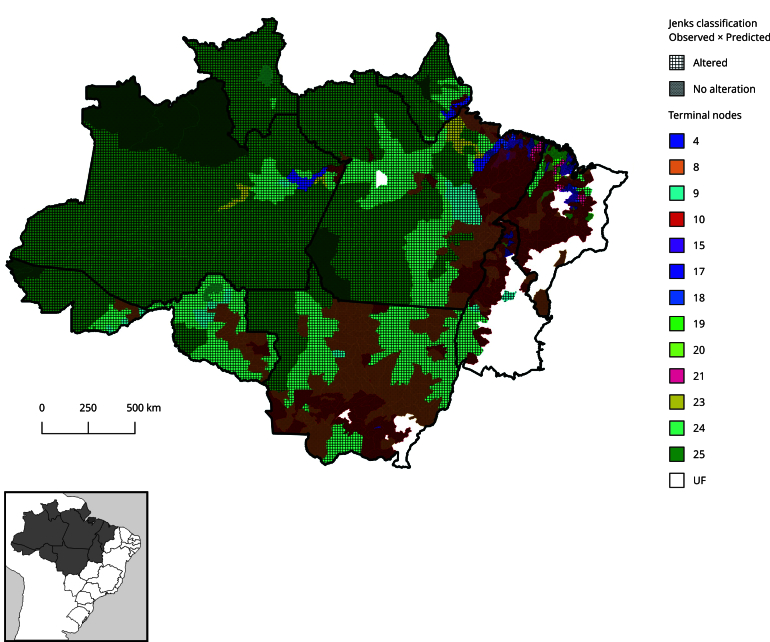
Spatial distribution of municipalities according to the final classification of nodes and the changes in incidence classes (low, moderate, and high) after analysis with the Conditional Inference Tree (CIT) model.

Regarding the group of municipalities associated with node 4, 11 municipalities were reclassified, moving to the category of moderate incidence of both malaria and Chagas disease. These municipalities include Colares, Mocajuba, Barcarena, Ananindeua, and Cametá in Pará; Bacurituba in Maranhão; Itacoatiara and Urucurituba in Amazonas; Igarapé-Miri in Pará; and Macapá in Amapá. Also, the clusters corresponding to nodes 9, 10, 15, and 17 did not show significant changes in the classifications of the municipalities, as illustrated in Figure 3.

UF: Federative Unit.

### Observed and predicted distribution of co-occurrence of Chagas disease and malaria

**Figure 4:**
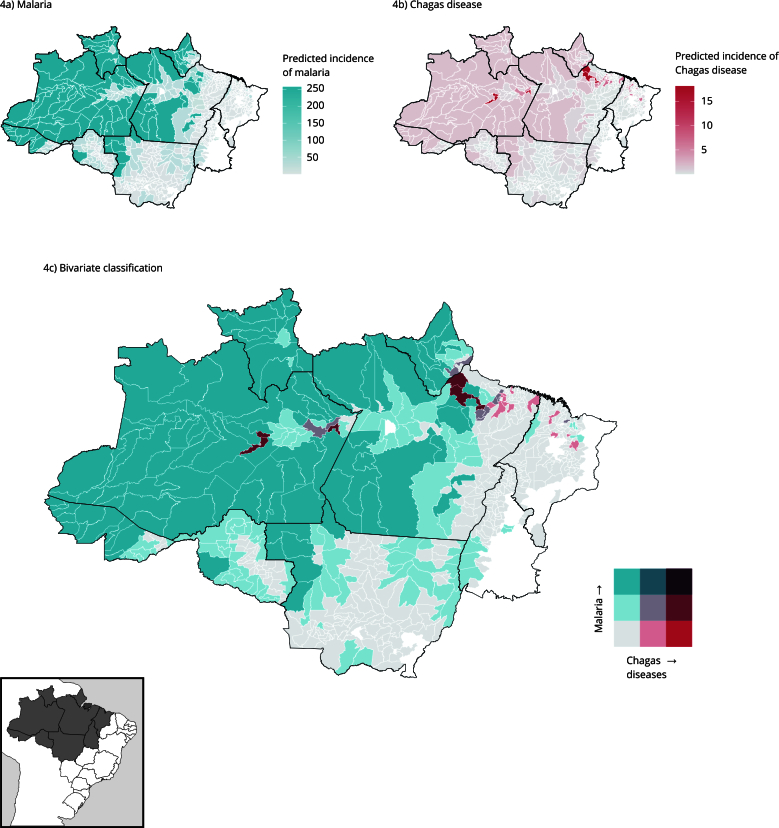
Conditional Inference Tree (CIT) model for incidence of malaria and Chagas disease, and bivariate classification between the two diseases by the Jenks method.

Note: the lower right-hand end of the legend in figure 4c (red) shows high rates of Chagas disease, while the upper left-hand end (blue) shows high rates of malaria, and the diagonal of the legend corresponds to the co-occurrence of the diseases.

Figures 4a and 4b show the predicted distributions of malaria and Chagas disease from 2015 to 2019, according to the socioenvironmental model. An increase is observed in the number of municipalities with a high incidence of malaria and Chagas disease when compared to the observed incidence. The same pattern is observed in Figure 4c in the distribution of Chagas disease and malaria using the Jenks method, particularly in the municipalities of Afuá, Breves, Limoeiro do Ajuru, and São Sebastião da Boa Vista in Pará, and Boa Vista dos Ramos in Amazonas State.

Table 1 shows the change in the number of municipalities before (the Jenks method) and after the analysis with the CIT model. At the end of the analysis, of all 652 municipalities analyzed, 231 (35%) showed a change in the classes. The low incidence class for both variables had 589 municipalities using the Jenks model, and after the ARIC model it decreased by 30% (409). The municipalities with a moderate classification for both diseases increased from 2 to 11, while the high incidence for Chagas disease and moderate incidence for malaria increased from 1 to 7, including Afuá, Limoeiro do Ajuru, Breves, and São Sebastião da Boa Vista in Pará State. The highest variation occurred in the low incidence for Chagas disease and high incidence for malaria, which increased from 8 to 106 municipalities.

**Table 1:** Comparison between the classification of municipalities according to the Jenks^23^ distribution method for incidence and for the values predicted in the Conditional Inference Tree (CIT) model.

Chagas disease	Malaria	Municipalities		Observed rate		Predicted rate	
		Observed	Predicted	Chagas disease	Malaria	Chagas disease	Malaria
Low	Low	589	409	0.0-5.1	0.0-199.6	0.0-2.0	0.0-2.8
Low	Low	31	105	0.0-3.9	217.4-732.0	0.2-0.8	18.8-32.5
Low	High	8	106	0.0-3.3	803.0-2083.6	1.8-1.8	257.1-257.1
Moderate	Low	14	14	8.6-21.6	0.2-113.9	3.3-7.1	0.4-0.6
Moderate	Moderate	2	11	10.4-12.7	248.8-290.2	5.3-5.3	20.6-20.6
Moderate	High	2	-	14.8-15.8	859.6-950.6	-	-
High	Low	4	-	27.0-47.6	20.7-156.8	-	-
High	Moderate	1	7	25.1-25.1	373.1-373.1	18.1-18.1	63.2-63.2
High	High	1	-	31.5-31.5	850.5-850.5	-	-
Total		652	652				

## Discussion

In the Amazon, malaria and Chagas disease are two common and persistent parasitic diseases, with heterogeneous distribution, which impose a significant burden on public health, with an impact on the economy of local communities ^2^. 

At first, our results identified four areas with a high incidence of malaria, in western Acre, northern Amazonas and Pará, and western Amapá. Each municipality has known characteristics that help increase the local burden of the disease, such as fish farming tanks without proper management in western Acre ^31^, precipitation and the influence of river levels in Amazonas and Pará states ^32^ ^,^ ^33^, mining and mineral exploration activities ^34^ ^,^ ^35^, piassava extraction in activities Amapá, and açaí production in the Brazilian Amazon ^35^ ^,^ ^36^. Regarding Chagas disease, some municipalities in Pará State showed a high incidence of the disease - this region that has reported high incidence rates of the disease mainly due to the production of açaí ^37^. In the predicted model, an expansion of the areas affected by both diseases was observed, showing the extent of the region with favorable conditions for the occurrence of both diseases. This fact demands attention of surveillance systems and action plans for the region, particularly considering the increasing deforestation and ongoing environmental transformations.

Our results identified municipalities in Pará with a high incidence of co-occurrence of malaria and Chagas disease. It highlights the importance of an integrated approach that takes into account existing diseases and the complexity of the factors involved, in order to develop effective strategies for detection, treatment, management, and broader interventions.

High vegetation cover was the main predictor for the occurrence of malaria. In municipalities in the Amazon, with moderate to high native forest cover, occupation occurs interspersed with the forest, an ideal condition for the reintroduction and introduction of known and unknown pathogens in the human population. Unlike Chagas disease, the occurrence was explained by different combinations of variables, so that the tree incorporates more branches. In other words, unlike malaria, whose occurrence in 16% of municipalities was explained by the variable core, for Chagas disease, a larger set of associated variables emerge as predictors, combining more urban contexts, but maintaining characteristics of rural landscapes and forest, suggesting more heterogeneous municipal profiles. Over the years, naturally infected triatomines have been frequently found in urban environments ^38^. In this scenario, it is important to adapt vector control programs to this new transmission context, taking into account the particular characteristics of the lifestyle in urban areas ^39^.

Our findings show that municipalities with pasture and moderate to high forest cover showed a positive association with the transmission of both diseases. In pasture areas, the presence of water bodies and palm trees of the genus Attalea − described as a natural habitat for populations of Rhodnius, vectors of Chagas − is common, used as a source of water and shade for livestock and fish farming tanks ^40^ ^,^ ^41^. 

The co-occurrence of Chagas disease and malaria is associated with a complex network of multifaceted factors, including environmental, socioeconomic, and cultural aspects ^42^. Studies suggest that in endemic areas, these factors play an important role in transmission and exposure and generate a cycle of perpetuated vulnerability, aggravating social and economic impacts, since diseases disproportionately affect marginalized populations ^43^ ^,^ ^44^. These diseases, when not treated properly, results in physical disabilities and long-term consequences that overburden public health systems, requiring continuous investment in monitoring and clinical management as well as adaptations in surveillance and control programs that take into account local socioeconomic realities ^43^.

Regarding study limitations, both the CIT method and the quality of data can compromise the results. For the CIT method, the following should be noted: sensitivity to small data variations, which can affect the stability and replicability of the model; the tendency to overfitting that affects performance of new data; challenges related to the interpretation of continuous variables; limitations in linear relationship modeling; the inability to extrapolate; and the potential bias for variables with many categories. Also, the model complexity increases with large volumes of data, requiring more computational resources. In terms of data, limitations include the need for harmonization due to territorial changes and the creation of new municipalities, the accuracy and omissions in PRODES deforestation data, underreporting of malaria and Chagas disease cases, aggregation in time and space, uncertainties in the attribution of disease cases between rural and urban areas in Brazilian Information System for Notificable Diseases (SINAN, acronym in Portuguese), and variations in the Census questionnaires, which affect the comparability and interpretation of trends, particularly in terms of the MPI and the capture of living conditions in rural Amazon ^21^. These limitations highlight the complexity of the challenges in data analysis and interpretation, suggesting the need for caution and methodological adjustments to better reflect the reality studied.

The co-occurrence of malaria and Chagas disease in the Amazon represents a challenge for public health, given the complexity of the factors that drive the expansion of these diseases. It highlights the need for integrated surveillance, using statistical and spatial analysis tools to improve management, treatment, and prevention strategies. Statistical models that consider multiple diseases help understand their interactions ^45^, while spatial analysis is crucial to identify vulnerable areas and intervention targets ^46^. The integration of these tools in epidemiological surveillance helps understand local determinants of health and contributes to the formulation of public policies that are more appropriate for each territorial context.
