# Supplementary material for: Co-occurrence of malaria and Chagas disease in the Brazilian Amazon: the need for integrated health surveillance
Source: Cad Saude Publica. 2025 Jun 9;41(Suppl 1):e00042124. doi: 10.1590/0102-311XEN042124 (PMC12161506; doi:10.1590/0102-311XEN042124)
Supplement: Supplementary file 1 [file 1678-4464-csp-41-s1-EN042124-s.pdf]

Supplementary Material

Table S1 Mean and confidence interval of the study variables (95%CI) for the municipalities classified in each terminal node of the regression tree with conditional inference.

		Terminal nodes												
		**4**	**8**	**9**	**10**	**15**	**17**	**18**	**19**	**20**	**21**	**23**	**24**	**25**
Malaria	95%CI	10.82-30.38	2.04-3.59	13.65-24.14	0.64-0.95	0.26-2	0.01-0.04	0.28-0.53	0.93-2.07	2.42-3.20	0.21-0.89	41.42-83.91	20.79-43.99	222.45-293.10
	Media	20.56	2.81	18.85	0.80	0.37	0.03	0.41	1.50	2.80	0.56	63.23	32.47	257.07
Chagas	95%CI	3.08-7.46	0.01-0.03	0.10-0.27	0.14-0.23	0.08-0.22	0.30-0.76	0.75-5.93	0.12-1.18	0.10-3.90	4.12-10.01	10.82-25.10	0.43-1.15	1.27-2.25
	Media	5.27	0.02	0.18	0.18	0.15	0.53	3.34	0.66	2.00	7.13	18.13	0.79	1.76
Core	95%CI	17.82-28.73	9.17-14.00	20.65-26.47	6.5-8.69	0.64-1.36	5.99-7.26	7.25-12.14	4.1-7.04	-0.1-0.42	0.07-11.2	60.9-77.22	53.95-56.75	86.51-88.22
	Media	24.01	16.58	21.34	11.75	1.16	7.56	10.21	6.33	0.55	9.78	60.73	56.4	86.06
Pasture	95%CI	0.84-1.47	48.72-58.85	37.9-49.86	45.52-49.46	29.32-40.31	13.54-24.83	11.13-25.5	17.85-24.87	53.74-56.34	14.91-36.32	-0.01-0.07	11.08-16.01	
	Media	1.34	53.51	43.61	45.81	32.71	21.24	15.15	23.66	55.48	21.71	0.07	14.42	
secveg	95%CI		8.73-9.13	7.34-9.57	20.97-22.36	47.21-55.07	40.57-44.4	41.84-49.13	42.95-50.87	38.85-43.16	39.45-44.9			
	Media		8.71	8.24	22.34	52.36	44.5	43.22	51.03	40.81	42.95			
edge	95%CI					47.82-59.51	50.39-60.38	48.23-64.9	44.14-51.58	24.56-34.51	103.16-116.51			
	Media					54.16	57.64	55.23	48.57	26.09	111.95			
deorg	95%CI		0.57-0.72	4.46-5.39										
	Media		0.84	5.81										
Imp_urb	95%CI					1.72-2.68	1.85-2.35	0.61-1.89	4.79-6.05					
	Media					2.42	2.33	1.79	5.79					
urban	95%CI						0.16-0.34	0.84-1.75						
	Media						0.35	1.53						

95%CI: 95% confidence interval.
Note: bootstrap (R = 1,000).

Table S2 Error analysis for predicted values in the ARIC model for Chagas disease.

Nodes	EQM	SD	N	SE	95%CI	Chagas_pred
4	6.14	3.74	11	1.13	4.56-8.80	5.27
8	0.04	0.11	147	0.01	0.03-0.07	0.02
9	0.29	0.23	18	0.05	0.21-0.46	0.18
10	0.32	0.56	199	0.04	0.26-0.43	0.18
15	0.23	0.20	19	0.04	0.17-0.36	0.15
17	0.79	0.69	20	0.15	0.58-1.21	0.53
18	4.71	5.30	7	2.00	2.23-9.09	3.34
19	1.08	1.84	16	0.46	0.59-2.72	0.66
20	3.50	4.24	8	1.50	2.00-6.50	2.00
21	8.05	1.58	7	0.60	7.21-9.60	7.13
23	17.56	7.26	7	2.74	12.91-23.44	18.13
24	1.39	3.22	87	0.35	0.92-2.59	0.79
25	2.65	4.27	106	0.41	2.05-3.96	1.76

MSE: mean square error; SD: standard deviation; SE: standard error.

Table S3 Error analysis for predicted values in the ARIC model for malaria.

Node	EQM	SD	N	SE	95%CI	Mal_pred
4	25.34	22.83	11	6.88	17.65-52.47	20.56
8	4.16	8.56	147	0.71	3.20-6.46	2.81
9	18.41	11.87	18	2.80	13.42-25.32	18.85
10	1.12	1.93	199	0.14	0.93-1.56	0.80
15	0.35	0.31	19	0.07	0.25-0.59	0.37
17	0.04	0.04	20	0.01	0.03-0.08	0.03
18	0.26	0.24	7	0.09	0.13-0.46	0.41
19	1.76	1.37	16	0.34	1.31-2.76	1.50
20	0.98	0.52	8	0.18	0.64-1.31	2.80
21	0.64	0.71	7	0.27	0.33-1.67	0.56
23	52.16	24.17	7	9.13	37.00-71.15	63.23
24	47.53	98.20	87	10.53	33.34-83.35	32.47
25	255.88	246.96	106	23.99	220.33-314.98	257.07

95%CI: 95% confidence interval; MSE: mean square error; SD: standard deviation; SE: standard error.
