# Supplementary material for: Co-occurrence of malaria and Chagas disease in the Brazilian Amazon: the need for integrated health surveillance
Source: Cad Saude Publica. 2025 Jun 9;41(Suppl 1):e00042124. doi: 10.1590/0102-311XEN042124 (PMC12161506; doi:10.1590/0102-311XEN042124)
Supplement: Supplementary file 2 [file 1678-4464-csp-41-s1-EN042124-s1.pdf]

Material Suplementar

Tabela S1 Média e intervalo de confiança das variáveis de estudo (IC95%) para os municípios classificados em cada nó terminal da árvore de regressão com inferência condicional.

		**Nós terminais**												
		**4**	**8**	**9**	**10**	**15**	**17**	**18**	**19**	**20**	**21**	**23**	**24**	**25**
Malária	IC95%	10,82-30,38	2,04-3,59	13,65-24,14	0,64-0,95	0,26-2	0,01-0,04	0,28-0,53	0,93-2,07	2,42-3,20	0,21-0,89	41,42-83,91	20,79-43,99	222,45-293,10
	Média	20,56	2,81	18,85	0,80	0,37	0,03	0,41	1,50	2,80	0,56	63,23	32,47	257,07
Chagas	IC95%	3,08-7,46	0,01-0,03	0,10-0,27	0,14-0,23	0,08-0,22	0,30-0,76	0,75-5,93	0,12-1,18	0,10-3,90	4,12-10,01	10,82-25,10	0,43-1,15	1,27-2,25
	Média	5,27	0,02	0,18	0,18	0,15	0,53	3,34	0,66	2,00	7,13	18,13	0,79	1,76
Core	IC95%	17,82-28,73	9,17-14,00	20,65-26,47	6,5-8,69	0,64-1,36	5,99-7,26	7,25-12,14	4,1-7,04	-0,1-0,42	0,07-11,2	60,9-77,22	53,95-56,75	86,51-88,22
	Média	24,01	16,58	21,34	11,75	1,16	7,56	10,21	6,33	0,55	9,78	60,73	56,4	86,06
Pasture	IC95%	0,84-1,47	48,72-58,85	37,9-49,86	45,52-49,46	29,32-40,31	13,54-24,83	11,13-25,5	17,85-24,87	53,74-56,34	14,91-36,32	-0,01-0,07	11,08-16,01	
	Média	1,34	53,51	43,61	45,81	32,71	21,24	15,15	23,66	55,48	21,71	0,07	14,42	
secveg	IC95%		8,73-9,13	7,34-9,57	20,97-22,36	47,21-55,07	40,57-44,4	41,84-49,13	42,95-50,87	38,85-43,16	39,45-44,9			
	Média		8,71	8,24	22,34	52,36	44,5	43,22	51,03	40,81	42,95			
edge	IC95%					47,82-59,51	50,39-60,38	48,23-64,9	44,14-51,58	24,56-34,51	103,16-116,51			
	Média					54,16	57,64	55,23	48,57	26,09	111,95			
deorg	IC95%		0,57-0,72	4,46-5,39										
	Média		0,84	5,81										
Imp_urb	IC95%					1,72-2,68	1,85-2,35	0,61-1,89	4,79-6,05					
	Media					2,42	2,33	1,79	5,79					
urban	IC95%						0,16-0,34	0,84-1,75						
	Média						0,35	1,53						

IC95%: intervalo de 95% de confiança.
Nota: *bootstrap* (R = 1000).

Tabela S2 Análise de erros de para valores preditos no modelo ARIC para doença de Chagas.

Nó	EQM	SD	N	SE	IC95%	Chagas_pred
4	6,14	3,74	11	1,13	4,56-8,80	5,27
8	0,04	0,11	147	0,01	0,03-0,07	0,02
9	0,29	0,23	18	0,05	0,21-0,46	0,18
10	0,32	0,56	199	0,04	0,26-0,43	0,18
15	0,23	0,20	19	0,04	0,17-0,36	0,15
17	0,79	0,69	20	0,15	0,58-1,21	0,53
18	4,71	5,30	7	2,00	2,23-9,09	3,34
19	1,08	1,84	16	0,46	0,59-2,72	0,66
20	3,50	4,24	8	1,50	2,00-6,50	2,00
21	8,05	1,58	7	0,60	7,21-9,60	7,13
23	17,56	7,26	7	2,74	12,91-23,44	18,13
24	1,39	3,22	87	0,35	0,92-2,59	0,79
25	2,65	4,27	106	0,41	2,05-3.96	1,76

IC95%: intervalo de 95% de confiança.
EQM: erro quadrático médio; SD: desvio padrão; SE: erro padrão.

Tabela S3 Análise de erros de para valores preditos no modelo ARIC para malária.

Nó	EQM	SD	N	SE	IC95%	Mal_pred
4	25,34	22,83	11	6,88	17,65-52,47	20,56
8	4,16	8,56	147	0,71	3,20-6,46	2,81
9	18,41	11,87	18	2,80	13,42-25,32	18,85
10	1,12	1,93	199	0,14	0,93-1,56	0,80
15	0,35	0,31	19	0,07	0,25-0,59	0,37
17	0,04	0,04	20	0,01	0,03-0,08	0,03
18	0,26	0,24	7	0,09	0,13-0,46	0,41
19	1,76	1,37	16	0,34	1,31-2,76	1,50
20	0,98	0,52	8	0,18	0,64-1,31	2,80
21	0,64	0,71	7	0,27	0,33-1,67	0,56
23	52,16	24,17	7	9,13	37,00-71,15	63,23
24	47,53	98,20	87	10,53	33,34-83,35	32,47
25	255,88	246,96	106	23,99	220,33-314,98	257,07

IC95%: intervalo de 95% de confiança.

EQM: erro quadrático médio; SD: desvio padrão; SE: erro padrão.
