# Supplementary material for: Co-occurrence of malaria and Chagas disease in the Brazilian Amazon: the need for integrated health surveillance
Source: Cad Saude Publica. 2025 Jun 9;41(Suppl 1):e00042124. doi: 10.1590/0102-311XEN042124 (PMC12161506; doi:10.1590/0102-311XEN042124)
Supplement: Translation

# Translation: Coocorrência de malária e doença de Chagas na Amazônia brasileira: a necessidade de uma vigilância em saúde integrada

**DOI:** 10.1590/0102-311XPT042124

**Authors:** Izabel Cristina dos Reis, Raquel Martins Lana, Cláudia Torres Codeço, Ana Paula Dal’Asta, Milton Barbosa, Diego Ricardo Xavier

**Affiliations:** 1 Instituto de Comunicação e Informação Científica e Tecnológica em Saúde, Fundação Oswaldo Cruz, Rio de Janeiro, Brasil.; 2 Centro Nacional de Supercomputación, Barcelona, España.; 3 Programa de Computação Científica, Fundação Oswaldo Cruz, Rio de Janeiro, Brasil.; 4 Instituto Nacional de Pesquisas Espaciais, São José dos Campos, Brasil.; 5 Laboratório de Ecologia Evolutiva e Biodiversidade, Universidade Federal de Minas Gerais, Belo Horizonte, Brasil.

**Keywords:** Malária, Doença de Chagas, Integralidade em Saúde, Árvores de Decisões

## Abstract

Este estudo aborda a coocorrência de malária e doença de Chagas nos municípios da Amazônia, região caracterizada por diversidade geográfica e climática e por transformações socioeconômicas e ambientais. O objetivo do estudo foi identificar os fatores relacionados à coocorrência de malária e doença de Chagas na Amazônia Brasileira no período entre 2015 e 2019. A análise explorou 19 indicadores ambientais e dois socioeconômicos relacionados à perda de habitat, ao uso e cobertura da terra, às anomalias climáticas e ao índice de pobreza multidimensional. A modelagem foi realizada por Árvores de Regressão com Inferência Condicional, ajustando modelos com e sem variáveis contextuais, para mapear áreas de provável coocorrência das doenças. A incidência de malária predomina na Amazônia ocidental, enquanto a doença de Chagas apresenta maior concentração em áreas do Pará e partes do Amazonas e Acre. Municípios com alta cobertura de vegetação nativa mostraram maiores incidências de malária, mas não necessariamente de doença de Chagas. Municípios com cobertura vegetal nativa e áreas de pastagem apresentaram uma heterogeneidade na incidência das doenças, com alguns apresentando alta incidência de ambas. A análise preditiva passou de 1 para 7 o número de municípios com alta incidência esperada de malária (média) e doença de Chagas (alta) em comparação com os dados observados. As análises indicam áreas com risco de média e alta incidência de ambas as doenças, cobrindo uma região maior do que a observada no período. Alternativas de vigilância compartilhada e a integração de programas para identificação de casos e tratamento podem ser um caminho para otimização de recursos e busca pela erradicação desses agravos na região.

## Introdução

A Amazônia brasileira é um território complexo e dinâmico composto por uma variedade de povos, paisagens e ecossistemas, além de englobar grande parte da floresta tropical amazônica, a maior reserva de diversidade de espécies biológicas do mundo, com vasta quantidade de recursos naturais e papel crucial na regulação dos padrões climáticos locais e regionais ^1^ ^,^ ^2^. Desde 2012, tem se observado um aumento progressivo nas taxas de desmatamento, com ápice em 2021, quando mais de 13 mil km^2^ de floresta amazônica foram suprimidos ^3^. Estima-se que aproximadamente metade (2,5 milhões de km^2^) da floresta esteja atualmente degradada ^4^.

Esse quadro tem influenciado direta e indiretamente a dinâmica das doenças transmitidas por insetos vetores, as quais causam um impacto significativo em termos socioeconômicos e de saúde na população da região, principalmente aquelas expostas a condições precárias de habitação e de trabalho ^5^. As alterações no ambiente tendem a mudar o balanço ecológico e o contexto dentro do qual patógenos, parasitas e seus vetores e hospedeiros se desenvolvem e transmitem as doenças ^6^.

Na Amazônia ocorre uma série de doenças transmitidas por vetores que afetam as populações locais, dentre elas, malária e doença de Chagas. A malária é uma das doenças infecciosas mais prevalentes na Amazônia, causada pelos parasitas do gênero *Plasmodium* e transmitida ao homem pela picada de fêmeas infectadas de mosquitos do gênero *Anopheles*. Entre os anos de 2013 e 2023 foram reportados 1.642,714 casos na Região Amazônica, a maioria causada pelo *P. vivax* (86 %) ^7^. *A. darlingi* é o principal vetor, mas outras espécies também são implicadas como vetores da malária na região ^8^. A dinâmica de transmissão da malária nas zonas de atividades econômicas extrativistas, nas zonas agrícolas, nas áreas urbanas e no perímetro suburbano e nas reservas indígenas são distintas, uma vez que envolvem populações humanas com características demográficas, sociais e econômicas específicas ^9^ e paisagens diferenciadas que determinam diferentes fatores ecológicos de respectivas comunidades de *Anopheles* ^10^. O desmatamento e o garimpo estão na listagem dos principais desafios do programa de eliminação da malária na Região Amazônica ^11^.

A doença de Chagas é uma enfermidade produzida por *Trypanosoma cruzi* e transmitida ao ser humano por triatomíneos dos gêneros *Triatoma*, *Rhodnius* e *Panstrongylu*s. No Brasil, a transmissão doença de Chagas pela ingestão do suco contaminado do açaí, processado artesanalmente sem tratamento térmico, tem sido cada vez mais relatada ^12^. O país notificou 2.777 casos agudos de doença de Chagas entre os anos de 2010 e 2020. A Região Norte registrou 84% desses casos, destacando-se o Estado do Pará com 1.996 casos agudos ^13^. A migração progressiva de pessoas e animais domésticos de áreas rurais para urbanas e a adaptação dos vetores às periferias das cidades devido ao deslocamento causado pelo desmatamento ou urbanização têm favorecido a manutenção do ciclo de transmissão da doença de Chagas, principalmente pela ingestão de alimentos contaminados ^14^ ^,^ ^15^.

A detecção de *T. cruzi* em lâminas de gota espessa de pacientes assintomáticos e sintomáticos para malária, uma estratégia implementada em 2008, tem aumentado a sensibilidade na detecção da ocorrência ou coocorrência e de coinfecções entre esses dois agravos ^16^ ^,^ ^17^. Essa sobreposição oferece possíveis sinergias e oportunidades para a utilização de recursos comuns para uma abordagem integrada de Saúde Pública para ambas as doenças. Nesse contexto, este estudo aborda a coocorrência de malária e doença de Chagas na mesma população, sem investigar a coinfecção entre seus agentes etiológicos, que apesar de representar um risco nesse cenário, ocorre em apenas 0,2% dos casos confirmados de malária ^7^. A coocorrência representa um desafio substancial para a saúde pública na Amazônia, demandando estratégias de vigilância, diagnóstico e controle que considerem as complexas interações ecológicas e sociais da região. A vigilância integrada é essencial nesse contexto, pois promove a colaboração de diferentes programas de controle, setores e comunidades, utilizando dados para monitorar e responder eficientemente a surtos e propagação de doenças ^18^. Portanto, o objetivo deste estudo foi identificar os fatores ambientais e sociais relacionados à coocorrência de malária e Chagas na Amazônia Brasileira com um olhar para vigilância integrada de saúde pública.

## Método

### Área de estudo

A Amazônia Legal, região político-administrativa ocupa aproximadamente 5 milhões de km^2^, representando cerca de 59% do território brasileiro. Essa região abrange nove estados distribuídos em diferentes regiões geográficas do país: Acre, Amapá, Amazonas, Pará, Rondônia, Roraima e Tocantins no Norte; parte do Maranhão no Nordeste; e parte do Mato Grosso no Centro-oeste. Estruturada em 772 municípios e com cerca de 18 milhões de habitantes ^19^, essa área possui uma diversidade geográfica e administrativa significativa.

Geograficamente posicionada na zona equatorial, a área de estudo é caracterizada por um clima predominantemente quente e úmido ^20^, marcado por um período chuvoso e um período de estiagem. A intensidade e a duração desses períodos pode variar em cada município/localidades.

### Dados e variáveis

Utilizou-se a base de dados harmonizados para a Amazônia Legal Brasileira denominada Trajetórias *dataset* ^21^ (https://zenodo.org/records/7098053). Essa base contém uma série de indicadores ambientais, epidemiológicos e socioeconômicos a nível municipal que cobre as duas primeiras décadas do século XXI. Para este estudo, foram utilizados dados referentes ao total de casos notificados de malária (*vivax*, *falciparum* e mista) e casos notificados de doença de Chagas aguda no período de 2015 a 2019, por zona rural e urbana de cada município. Os casos de malária para toda espécie e de ambas as zonas foram agregados para obter o total de casos por município no período de cinco anos. O tamanho populacional utilizado para o cálculo dos indicadores epidemiológicos foi aquele referente ao ano de 2017, disponível no Trajetórias *dataset*. A partir do total de casos, foi calculado o índice parasitário anual (IPA) para o período analisado de cada município, como descrito em Rorato et al. ^21^. O IPA é o indicador mais utilizado para malária e mede o número de exames positivos da doença por município dividido pela população do município no período do estudo, multiplicado por 1.000. Não é uma medida de incidência porque há incerteza na atribuição de um novo caso ou recaída por malária *vivax* ^22^, contudo, para efeitos de comparação com a doença de Chagas, o chamaremos de incidência. A incidência de Chagas foi calculada usando a mesma expressão matemática, tendo o total de casos novos agudos no numerador e o mesmo denominador utilizado para a malária.

Para identificar fatores ambientais associados com a coocorrência das duas doenças, foram utilizados 19 indicadores ambientais presentes no Trajetórias *dataset*. Desses, sete descrevem a perda de *habitat* observada entre 2010 e 2017 em decorrência da perda de cobertura florestal, ou seja, desmatamento do período em relação a floresta original (*deorg*) e em relação ao remanescente florestal no início do período (*defor*), fogo florestal (*fire*) e a perda gradual de floresta por corte seletivo e/ou fogo (degradação da floresta original - *dgorg*; e degradação recente - *dgfor*), além de duas medidas de fragmentação espacial das áreas com cobertura de vegetação natural relativas à área núcleo (*core*) e borda (*edge*). Outros seis indicadores descrevem as pegadas deixadas na paisagem pelos sistemas produtivos, medidas a partir de classes de uso e cobertura da terra mapeadas em 2017 proporção de pastagem (*pasture*), de floresta remanescente (*refor*), de vegetação secundária (*secveg*), de agricultura (*crop*), de mineração (*mining*), e de área urbanizada (*urban*). Três indicadores ainda descrevem a mobilidade e a conectividade medindo a densidade das redes de transporte em 2017 fluviais (hidrovias - *river*; e portos - *ports*) e terrestres (*roads*). E, por fim, três medidas da magnitude espacial dos eventos de anomalias climáticas no período de 2007 a 2017, referentes à precipitação (*precp* - anomalia positiva; *precn* - anomalia negativa) e à temperatura (*tempp* - anomalia de temperatura positiva). Dos indicadores socioeconômicos disponíveis na base de dados, utilizou-se apenas o índice de pobreza multidimensional (IPM) construído para 2010 para os estratos rural (*ipm_rural*) e urbano (*ipm_urb*) de cada município. O IPM é uma combinação da proporção da população vivendo em uma condição de pobreza multidimensional pela intensidade dessa pobreza nas dimensões de saúde, educação e condições de vida. Todos esses 21 indicadores foram utilizados sem processamentos adicionais. Parte dos indicadores ambientais presentes no Trajetórias *dataset* foram calculados a partir dos dados do Projeto de Monitoramento do Desmatamento na Amazônica Brasileira por Satélite (PRODES), que monitora o corte raso em áreas de fitofisionomia florestal primária na Amazônia Legal. Assim, para os municípios que mesmo incluídos na Amazônia Legal, mas com fitofisionomia diversa da florestal, não foram calculados os indicadores associados a essa cobertura (*deorg*, *defor*, *dgorg* e *dgfor*). Detalhes sobre a construção e o cálculo de cada indicador são encontrados em Rorato et al. ^21^.

### Análises dos dados

#### Identificação das áreas de coocorrência

Para identificar as áreas de coocorrência de notificação de malária e doença de Chagas na Amazônia Legal, foi realizado mapeamento bivariado pelo método de Jenks ^23^ utilizando a incidência das duas doenças como desfechos. Esse método é frequentemente aplicado na análise de dados geográficos, para agrupar dados espaciais de maneira que minimize a variação dentro de cada grupo e maximize a variação entre grupos. Para este estudo, foi considerada a distribuição de Jenks ^23^ em três dimensões (alto, médio e baixo) para os dois agravos analisados. O cruzamento entre essas geraram nove classes, representadas numa matriz cuja diagonal representa incidência baixa para ambas as doenças na posição inferior esquerda e incidência alta na posição superior direita (ver legenda da Figura 1). A análise foi realizada com a biblioteca *{biscale}* ^24^ software R (http://www.r-project.org).

**Figura 1:** Incidência de malária e doença de Chagas no período de 2015 a 2019, em municípios da Amazônia brasileira, e a classificação bivariada das duas doenças pelo método Jenks.

Nota: a legenda da Figura 1c indica em tons de vermelho, a incidência de doença de Chagas e em tons de azul, a incidência de malária. As cores da diagonal indicam gradiente de coocorrência.

#### Modelagem sócioambiental

Para mapear áreas de provável coocorrência das duas doenças (variáveis de desfecho), foram ajustados modelos de classificação multivariados, o primeiro sem covariáveis e o segundo modelo com as variáveis contextuais ambientais e sociais. O modelo sócioambiental de classificação é do tipo Árvore de Regressão com Inferência Condicional (ARIC), com três níveis primários de resultados consistindo em nó raiz, nós internos e nós terminais (folhas) ^25^. O modelo está implementado na biblioteca *{partykit::ctree()}* ^26^ no software R. Na epidemiologia, o ARIC tem se mostrado adequado para lidar com conjuntos de dados complexos e identificar interações entre múltiplos fatores que afetam a saúde da população ^27^. O ARIC faz uso de uma técnica de aprendizado de máquina não-paramétrica que utiliza o critério de partição baseado em significância estatística e avalia as relações condicionais entre as variáveis contextuais em busca de grupos homogêneos que descrevam a distribuição da variável de estudo. O algoritmo não apresenta viés na seleção de variáveis e é capaz de lidar tanto com dados numéricos quanto categóricos ^28^.

O algoritmo inicia testando a hipótese nula global de independência entre qualquer uma das variáveis contextuais e a variável de desfecho; caso a hipótese não possa ser rejeitada, a partição é interrompida. Se essa hipótese não puder ser rejeitada, a covariável que apresenta a associação mais forte com o desfecho de interesse é selecionada como candidata à divisão, por meio de testes de hipóteses estatísticos e seu valor de p. Se o valor de p mínimo for maior do que o limite de significância ajustado para múltiplos testes, nenhuma variável é selecionada para divisão e o nó é declarado como um nó terminal ^26^ ^,^ ^29^.

Neste estudo, dois parâmetros são destacados para construção do modelo socioambiental. O parâmetro *testtype* simulações de Monte Carlo para calcular a distribuição do teste estatístico que considera a soma dos quadrados dos resíduos e foi utilizado para avaliar se há associação significativa entre as covariáveis socioambientais e a incidência de malária e doença de Chagas). O parâmetro *mincriterion* determina o limiar de significância para uma divisão na ARIC, e foi estabelecido em 0,99 (valor de p < 0,001) o que resultou em uma ARIC mais conservadora com menos participações. A estatística de teste gerada por meio das simulações de Monte Carlo é então comparada ao *mincriterion* para decidir se uma divisão deve ser realizada. Com base nesses resultados, as partições foram realizadas e os grupos foram criados com os valores preditos das variáveis desfecho para os nós terminais. O cálculo de médias e intervalo de confiança das variáveis explicativas e os erros quadráticos médios (EQM) dos valores preditos nos grupos (nós terminais) são apresentados no Material Suplementar (https://cadernos.ensp.fiocruz.br/static//arquivo/suppl-e00042124-port_1071.pdf) e foram realizados através do método não-paramétrico *bootstrap* com parâmetro de reamostragem igual a 1.000 utilizando a biblioteca *{boot}* ^30^. Por fim, os valores estimados pelo modelo socioambiental foram novamente plotados utilizando a função bivariada com a distribuição de Jenks para mapeamento do risco predito de coocorrência de malária e doença de Chagas.

Para todas as análises, foram considerados apenas os municípios que apresentavam dados completos em todos os indicadores, resultando em 652 municípios (84,5%), de um total de 772. Os municípios excluídos são aqueles criados recentemente ou que não apresentaram dados para cálculo de alguns indicadores ambientais ^21^.

## Resultados

A Figura 1 apresenta a distribuição das incidências de malária e doença de Chagas no período de 2015 a 2019 nos 652 municípios estudados da Amazônia brasileira e a distribuição espacial conjunta considerando a distribuição de Jenks das duas variáveis. A malária ocorreu com maiores incidências principalmente na Amazônia Ocidental, nos municípios de Mâncio Lima, Rodrigues Alves e Cruzeiro do Sul no Acre; São Gabriel da Cachoeira, Barcelos e Santa Isabel do Rio Negro no Amazonas. Na Amazônia Oriental, a malária se concentrou em Anajás, Bagre e Jacareacanga no Pará e Serra do Navio e Calçoene no Amapá (Figura 1a). A doença de Chagas, por sua vez, parece ter uma distribuição mais concentrada, com as maiores incidências no nordeste Paraense, com destaque para os municípios de Limoeiro do Ajuru, Breves, Bagre, São Sebastião da Boa Vista e Muaná; e alguns municípios no Amazonas e Acre (Figura 1b).

Quando analisado o perfil de coocorrência pelo método Jenks (Figura 1c), apenas um município (Bagre, Pará) foi classificado como alto para as duas doenças. Dentre os municípios com média a alta coocorrência, observa-se um município (Curralinho, Pará) classificado como alto para doença de Chagas e médio para malária e dois municípios (Oeiras e Anajás, Pará) classificados como altos para malária e médios para doença de Chagas. Esses quatro municípios se encontram na Ilha de Marajó (Curralinho e Anajás) e na região do Baixo Rio Tocantins (Oeiras e Bagre, Pará). Dentre os municípios com incidência média para ambas as doenças, observam-se Carauari e Uarini, ambos pertencentes à região geográfica de Tefé no Amazonas. Por fim, observou-se 18 municípios (13 municípios no Pará, dois no Acre e no Maranhão e um no Amazonas) com atividade média (n = 14) a alta (n = 4) de doença de Chagas, mas baixa de malária; e 39 municípios (17 municípios no Amazonas, 11 em Roraima, seis no Amapá, três no Acre e um no Pará e em Rondônia) com atividade média (n = 31) a alta (n = 8) de malária, mas baixa de doença de Chagas.

O modelo ARIC ajustado manteve sete variáveis explicativas para o nível de coocorrência das duas doenças. Dentre as variáveis ambientais, são importantes preditores *core*, *pasture*, *edge*, *urban* e *secveg*. Dentre as variáveis de transformação florestal, o *deorg* se mostrou uma variável importante, e dentre os indicadores socioeconômicos, ficou o *ipm_urb*.

### Municípios com alta cobertura de vegetação nativa

A Figura 2 ilustra a saída gráfica do modelo ARIC. Trata-se de uma árvore com 13 nós finais, dos quais três agregam municípios com malária: 25, 23 e 24. A principal variável preditora para malária foi a cobertura florestal alta. Municípios com área *core* > 70,6% apresentaram as maiores incidências de malária. Essa variável mostrou uma relação direta com a ocorrência da doença, sendo responsável por classificar 16,3% dos municípios (nó 25), identificando municípios como Cruzeiro do Sul, Rodrigues Alves e Mâncio Lima, no Acre, e São Gabriel da Cachoeira e Barcelos, no Amazonas, todos na Amazônia Oriental. Em relação à doença de Chagas, também estão incluídos no nó 25 os municípios de Curralinho e Anajás, na ilha de Marajó e Bagre, no Baixo Tocantins no Pará, que haviam sido identificados pelo método Jenks com altas taxas tanto para doença de Chagas quanto para malária. Contudo, à exceção de alguns municípios, a maioria dos municípios nessa categoria de alta vegetação não relatam alta incidência de doença de Chagas.

**Figura 2:** Classificação dos municípios segundo o modelo Árvore de Regressão com Inferência Condicional (ARIC) e distribuição espacial dos nós finais para variáveis do projeto Trajetórias *dataset*, Amazônia brasileira.

### Municípios com cobertura de vegetação nativa moderada

A segunda variável preditora mais importante foi a área do município coberta por pastagem. Em 94 municípios (13,3%) com valores intermediários a altos de cobertura vegetal nativa (área core entre 43% e 70,6% da área municipal), a variável *pasture* foi importante para identificar a coocorrência de malária e Chagas. Ausência de pasto, definida aqui como *pasture* < 0,2%. esteve fortemente associada com a presença de doença de Chagas (nó 23). Essa condição foi encontrada em sete municípios (1,7%), dentre eles, Limoeiro do Ajuru e Breves no Estado do Pará, classificados como de incidência média para malária no método Jenks. Nos 87 municípios restantes, que possuem presença de pasto (*pasture* > 0.2%), encontra-se uma maior heterogeneidade de incidências de ambas as doenças (nó 24). Contudo, estão identificados nesse nó alguns municípios importantes com altas incidências em ambas as doenças, como Oeiras do Pará (Pará), e municípios com alta incidência de malária, em Cantá (Roraima) e Candeias do Jamari (Rondônia), e alta incidência para doença de Chagas, em Muaná (Pará) e Barreirinhas (Amazonas). Além disso, algumas capitais como Porto Velho (Rondônia) e Manaus (Amazonas) também estão classificadas no nó 24.

### Municípios com cobertura de vegetação nativa baixa

Em municípios com menor cobertura por vegetação nativa (*core* < 43%), a ocorrência de malária é ausente ou baixa. Nessas condições, a ocorrência da doença de Chagas está em geral associada à pouca cobertura de pastagem (< 2,4% do território) (nó 4). Nesse grupo, a maior incidência de malária foi observada em Cametá (Pará), cuja incidência para Chagas é considerada média. Já a maior incidência de Chagas é observada em Igarapé-Miri (Pará), com baixa incidência de malária. Ambos os municípios são vizinhos e localizados na região do Baixo Tocantins.

No restante dos municípios, caracterizados pela maior presença de pastagem (*pasture* > 2,4%), sete se destacam quanto à média incidência da doença de Chagas (nó 21). Esses possuem paisagens heterogêneas, onde a vegetação natural remanescente se encontra muito fragmentada (área *core* < 46% e *edge* > 90.514), com significativa cobertura de vegetação secundária (> 35%) e pastagens compondo mais de 2,4% da área municipal. Os municípios com maiores incidências nesse grupo são Acará, Bujaru e São Domingos do Capim, todos no Pará.

### Municípios com paisagem heterogênea

A inclusão de outras variáveis (*defor*, *deorg*, *urban* e *ipm-urban*) permitiu discriminar alguns perfis ambientais mais diversos associados à presença de doença de Chagas. Os nós 8, com 147 municípios (22,5%), e 9, com 18 municípios (2,8%), incluem a variável *deorg*, que mede o desmatamento em relação à área originalmente coberta por floresta. Com ponto de corte em 3,1%, são classificados municípios com baixa incidência de malária e doença de Chagas e muitos sem casos no período, grande parte no Mato Grosso, Maranhão, Tocantins e Pará. Os nós 15, 17, 18 e 19 incluem 62 municípios (9,5%) e são definidos pelas variáveis *ipm_urb* e *urban*, a primeira se refere a incidência da pobreza multidimensional urbana e a segunda da abrangência das áreas urbanizadas. Esses municípios apresentaram baixas incidências de malária, contudo cabe destacar a classificação do Município de Abaetetuba no Pará (nó 18) e Turilândia no Maranhão (nó 19), que possuem taxa elevada de doença de Chagas.

Considerando os agrupamentos delineados no nó 25, observa-se que, dos 106 municípios classificados, 98 apresentaram variações ao comparar a classificação obtida pelo método de Jenks com aquela resultante da aplicação do modelo ARIC. Tais variações indicam a influência significativa das variáveis preditoras. Dentre os municípios que conservaram suas classificações em ambas as análises, destacam-se Rodrigues Alves, Cruzeiro do Sul, Mâncio Lima no Acre, Barcelos, São Gabriel da Cachoeira e Santa Isabel do Rio Negro no Amazonas e Jacareacanga no Pará, os quais se mantiveram categorizados com alta incidência de malária e baixa incidência de doença de Chagas. Por outro lado, no nó 24, Candeias do Jamari (Rondônia) e Cantá (Roraima) permaneceram no grupo caracterizado por baixa incidência de Chagas e média incidência de malária. Constatou-se que a maioria dos demais municípios foi previamente classificada com baixas incidências para ambas as doenças.

No que concerne ao grupo de municípios associados ao nó 4, identificou-se que 11 sofreram reclassificação, passando para uma categoria de média incidência tanto de malária quanto de doença de Chagas. Esses municípios incluem Colares, Mocajuba, Barcarena, Ananindeua e Cametá no Pará, Bacurituba no Maranhão, Itacoatiara e Urucurituba no Amazonas. Igarapé-Miri no Pará, Macapá no Amapá. Adicionalmente, os agrupamentos correspondentes aos nós 9, 10, 15 e 17 não evidenciaram alterações significativas nas classificações dos municípios pertencentes, conforme ilustrado na Figura 3.

**Figura 3:** Distribuição espacial dos municípios segundo a classificação final dos nós e a alteração nas classes de incidência (baixa, média e alta) após a análise com o modelo Árvore de Regressão com Inferência Condicional (ARIC).

UF: Unidades da Federação.

### Distribuição observada e predita de coocorrência de doença de Chagas e malária

As Figuras 4a e 4b apresentam as distribuições preditas de malária e Chagas no período de 2015 a 2019, de acordo com o modelo socioambiental. Observa-se um aumento no número de municípios com alta incidência de malária e doença de Chagas comparado à incidência observada. O mesmo padrão se observa na Figura 4c na distribuição de doença de Chagas e malária pelo modelo de Jenks, com destaque para os municípios de Afuá, Breves, Limoeiro do Ajuru e São Sebastião da Boa Vista no Pará e Boa Vista dos Ramos no Amazonas.

**Figura 4:** Modelo Árvore de Regressão com Inferência Condicional (ARIC) para incidência de malária e doença de Chagas, e classificação bivariada entre as duas doenças pelo método Jenks*.*

Nota: na extremidade inferior direita da legenda da figura 4c (vermelho), observa-se altas taxas para doenças de Chagas, enquanto a extremidade superior esquerda (azul) apresenta altas taxas para malária, e a diagonal da legenda corresponde à coocorrência dos agravos.

Na Tabela 1, verifica-se a alteração no número de municípios antes (modelo Jenks) e após a análise com o modelo ARIC. No final da análise, dos 652 municípios analisados, 231 (35%) apresentaram alteração nas classes. A classe incidência baixa para as duas variáveis apresentava 589 municípios pelo modelo Jenks, e após o modelo ARIC, diminuiu em 30% (409). Os municípios com classificação média para ambos os agravos passaram de dois para 11, enquanto a classe alta para doença de Chagas e médio para malária subiram de um para sete, com destaque para Afuá, Limoeiro do Ajuru, Breves e São Sebastião da Boa Vista no Pará. A maior variação ocorreu na classe baixo para doença de Chagas e alto para malária, passando de oito para 106 municípios.

**Tabela 1:** Comparação entre a classificação dos municípios segundo a distribuição de Jenks ^23^ para a incidência e para os valores preditos pelo modelo Árvore de Regressão com Inferência Condicional (ARIC).

Doença de Chagas	Malária	Municípios		Taxa observada		Taxa predita	
		Observado	Predito	Doença de Chagas	Malária	Doença de Chagas	Malária
Baixo	Baixo	589	409	0,0-5,1	0,0-199,6	0,0-2,0	0,0-2,8
Baixo	Médio	31	105	0,0-3,9	217,4-732,0	0,2-0,8	18,8-32,5
Baixo	Alto	8	106	0,0-3,3	803,0-2083,6	1,8-1,8	257,1-257,1
Médio	Baixo	14	14	8,6-21,6	0,2-113,9	3,3-7,1	0,4-0,6
Médio	Médio	2	11	10,4-12,7	248,8-290,2	5,3-5,3	20,6-20,6
Médio	Alto	2	-	14,8-15,8	859,6-950,6	-	-
Alto	Baixo	4	-	27,0-47,6	20,7-156,8	-	-
Alto	Médio	1	7	25,1-25,1	373,1-373,1	18,1-18,1	63,2-63,2
Alto	Alto	1	-	31,5-31,5	850,5-850,5	-	-
Total		652	652				

## Discussão

Na Amazônia, malária e doença de Chagas são duas doenças parasitárias comuns e persistentes, com distribuição heterogênea, que impõem uma carga significativa para a saúde pública impactando a economia das comunidades locais ^2^.

Nossos resultados a princípio identificaram quatro áreas com alta incidência de malária, nas porções à oeste do Acre, norte do Amazonas e do Pará e oeste do Amapá. Cada município apresenta características conhecidas que contribuem para o aumento da carga local da doença, como: os tanques de piscicultura sem o devido manejo, a oeste do Acre ^31^; a precipitação e a influência do nível dos rios no Amazonas e Pará ^32^ ^,^ ^33^; as atividades de garimpo e exploração mineral ^34^ ^,^ ^35^; e as atividades de extração de piaçaba no Amapá e produção de açaí na Amazônia brasileira ^35^ ^,^ ^36^. Com relação à doença de Chagas, nossos achados destacaram alguns municípios no Pará com alta incidência da doença, região que ao longo do tempo vem se destacando com elevados índices, principalmente pelo consumo do açaí ^37^. No modelo predito, observou-se uma ampliação das áreas afetadas por ambas as doenças, evidenciando a extensão da região com condições propícias para a sua ocorrência. Esse fato demanda atenção nos sistemas de vigilância e nos planos de ações para a região, especialmente diante do crescente desmatamento e das transformações ambientais em curso.

Nossos resultados identificaram municípios do Pará com coocorrência de malária e doença de Chagas com alta incidência. Diante disso, reforça-se a necessidade de uma abordagem integrada que considere as doenças existentes e a complexidade dos fatores envolvidos, desenvolvendo estratégias eficazes para controle, detecção, tratamento e intervenções mais amplas.

A cobertura vegetal alta foi a principal preditora para a ocorrência de malária. Nos municípios da Amazônia, com presença de cobertura florestal nativa de moderada a elevada, a ocupação ocorre entremeada com a floresta, condição ideal para a reintrodução e introdução de patógenos conhecidos e desconhecidos na população humana. Diferentemente para doença de Chagas, a ocorrência foi explicada por combinações diversas de variáveis, de modo que a árvore incorpora mais ramificações. Ou seja, diferente da malária, cuja ocorrência em 16% dos municípios foi explicada pela variável core, para doença de Chagas, um conjunto maior de variáveis em combinação emergem como preditoras, combinando contextos mais urbanos, mas mantendo características de paisagens rurais e floresta, sugerindo perfis municipais mais heterogêneos. Ao longo dos anos, frequentemente têm sido encontrados triatomíneos infectados naturalmente em ambientes urbanos ^38^. Nesse cenário, ressalta-se a importância de adaptações dos programas de controle do vetor a esse novo contexto de transmissão, levando em consideração as características particulares do estilo de vida nos aglomerados urbanos ^39^.

Nossos achados mostram que municípios com pastagem e cobertura florestal de moderada a elevada apresentaram associação positiva com a transmissão de ambas as doenças. Nas áreas de pastagem é comum a presença de corpos d’água e de palmeiras do gênero *Attalea* - descritas como habitat natural para populações de *Rhodnius*, vetores de doença de Chagas - servindo como fonte de água e sombra para o gado, além de tanques de piscicultura ^40^ ^,^ ^41^.

A ocorrência de doença de Chagas e malária está associada a uma complexa rede de fatores multifacetados, incluindo os aspectos ambientais, socioeconômicos e culturais ^42^. Estudos apontam que em áreas endêmicas, esses fatores desempenham um papel fundamental na transmissão, exposição e geram um ciclo de perpetuação da vulnerabilidade, agravando os impactos sociais e econômicos, visto que as doenças afetam de maneira desproporcional as populações marginalizadas ^43^ ^,^ ^44^. A carga dessas doenças, quando não tratadas adequadamente, resulta em incapacidades físicas e consequências a longo prazo que sobrecarregam os sistemas de saúde pública, exigindo investimentos contínuos em monitoramento e manejo clínico, além de adaptações nos programas de vigilância e controle que levem em conta as realidades socioeconômicas locais ^43^.

Quanto às limitações do estudo, cabe ressaltar que tanto o método de árvores de regressão com inferência condicional quanto a qualidade dos dados utilizados podem comprometer os resultados. No que tange ao método, destacam-se a sensibilidade a pequenas variações nos dados, que pode afetar a estabilidade e a replicabilidade do modelo, a tendência ao sobreajuste que compromete o desempenho em novos dados, dificuldades na interpretação de variáveis contínuas, limitações na modelagem de relações lineares, a incapacidade de extrapolação e o potencial viés para variáveis com muitas categorias. Adicionalmente, a complexidade do modelo aumenta com grandes volumes de dados, exigindo mais recursos computacionais. Quanto aos dados, as limitações incluem a necessidade de harmonização devido a alterações territoriais e a criação de novos municípios, a precisão e as omissões nos dados de desmatamento do projeto PRODES, as subnotificações dos casos de malária e doença de Chagas, a agregação no tempo e no espaço e incertezas na atribuição de casos de doenças entre zonas rurais e urbanas no Sistema de Informação de Agravos de Notificação (SINAN), e as variações nos questionários do *Censo Demográfico*, que afetam a comparabilidade e a interpretação de tendências, particularmente no que se refere ao IPM e à captura das condições de vida na Amazônia rural ^21^. Essas limitações evidenciam a complexidade dos desafios enfrentados na análise e na interpretação dos dados, sugerindo a necessidade de cautela e ajustes metodológicos para melhor refletir a realidade estudada.

A coocorrência de malária e doença de Chagas na Amazônia representa um desafio para a saúde pública, dada a complexidade dos fatores que impulsionam a expansão dessas doenças. Isso enfatiza a necessidade de uma vigilância integrada, utilizando ferramentas de análise estatística e espacial para aprimorar estratégias de controle, tratamento e prevenção. Modelos estatísticos que consideram múltiplas doenças ajudam a entender suas interações ^45^, enquanto a análise espacial é crucial para identificar áreas vulneráveis e focos de intervenção ^46^. A integração dessas ferramentas na vigilância epidemiológica facilita a compreensão dos determinantes locais da saúde e contribui para a formulação de políticas públicas mais adequadas a cada contexto territorial.
